# A Roadmap for Computational Modelling of M/EEG

**DOI:** 10.1007/s10548-022-00889-x

**Published:** 2022-01-27

**Authors:** Benedetta Franceschiello, Jérémie Lefebvre, Micah M. Murray, Katharina Glomb

**Affiliations:** 1 EEG CHUV-UNIL Section, CIBM Center for Biomedical Imaging, Lausanne, Switzerland; 2 Laboratory for Investigative Neurophysiology, Department of Radiology, Lausanne University Hospital and University of Lausanne (CHUV-UNIL), Lausanne, Switzerland; 3 The Sense Innovation and Research Center, Lausanne and Sion, Switzerland; 4 Department of Biology, University of Ottawa, Ottawa, Canada; 5 Krembil Research Institute, University Health Network, Toronto, Canada; 6 Department of Mathematics, University of Toronto, Toronto, Canada; 7 Berlin Institute of Health at Charité; Department of Neurology with Experimental Neurology, Brain Simulation Section, Charité Universitätsmedizin Berlin, corporate member of Freie Universität Berlin and Humboldt-Universität zu Berlin, Berlin, Germany

This special issue is the result of ideas developed before, during and after a workshop^1^ that took place at the 2019 meeting of the Organization for Computational Neuroscience held in Barcelona. We, the guest editors, are very pleased that so many of the speakers agreed to author papers. We are also pleased that additional colleagues at different career stages have contributed original articles, opinions and insights to this special issue. We would like to thank all the reviewers that contributed their invaluable insights to ensuring the rigor and quality of the contributions to this special issue.

The motivation behind this special issue is our observation that computational modelling in EEG is not a particularly well-developed topic: the *in vitro* simulation of EEG signals is missing a clear inference of the neural mechanisms that generate the underlying brain activity at the microscale as well as a causal link between localized sources and observed behavior. EEG has much to gain from mechanistic models, as the plethora of dynamical phenomena that EEG offers would become more interpretable in terms of where they are generated and how they propagate. Beyond basic research, missing modelization puts serious limitations on how helpful EEG can be in the development of neurotechnology and clinical biomarkers: For example, while EEG is being routinely used in epilepsy wards around the world, the most important “tool” is still the expert clinician who identifies abnormal activity “by hand”, partly because no computational model in EEG offers, at present, enough added value.

We believe that in order to foster the integration of EEG with other neuroimaging modalities and to improve the development of usable clinical tools such as reliable biomarkers, we need a better understanding of the mechanisms underlying observed phenomena in M/EEG data. Computational models serve this purpose. Therefore, in this special issue, we called for and accepted papers that covered micro- (Saponati et al. 2021; Kohl et al. 2021), meso- (Byrne et al. 2021) and macroscale modelling of M/EEG dynamics (Allouch et al. 2021; Qin et al. 2021), as well as articles that show how models in M/EEG are applied today to improve data quality (Billings et al. 2021; Bhutada et al. 2021) as well as in a clinical context (Hutt and Lefebvre 2021; Sancristóbal et al. 2021). Two more articles (Glomb et al. 2021; Orczyk and Kajikawa 2021) summarize and provide a perspective on some aspects of the burgeoning field.

While reviewing and editing the articles, a speculative picture emerged of a roadmap of M/EEG modelling for the next few years. First, a greater standardization of M/EEG data analysis is necessary. What we need are easy(-ish)-to-use pipelines that give us the highest possible resolution and precision in our source localization and best fidelity in our functional connectivity measures. Second, with standardization, it will be easier to validate modelling results with empirical data. Here, integrating EEG with other neuroimaging modalities will likely play an important role as analysis and modelling approaches become more readily applicable to EEG. Third, through modelling, we will obtain a more precise characterization of the fast dynamics inherent in the M/EEG signal, and thus, greater access to the time scale at which many clinically and functionally relevant behaviors take place, opening the door to a new generation of reliable biomarkers.

Of course—many unforeseen roadblocks will reveal themselves on this journey, which in fact has already begun, and in a most non-linear fashion. We are merely proposing one of many possible perspectives on the present collection of articles, and in the remaining few paragraphs, we will lay out this perspective a little more clearly.

## Standardization—No “One Pipeline to Rule Them All”

Spurious variability introduced into the data by different (pre-)processing pipelines is, apart from the obvious problems with reproducibility of results, a major pitfall for modelers. This is why standardization is a necessary step to making M/EEG models more useful: A model needs to reproduce phenomena that do not depend on the preprocessing pipeline or the software used. The modeler needs to be sure that what they are explaining is not an artefact of the analysis pipeline, but actually attributable to brain activity.

While there are definite recommendations that can be made from a signal processing point of view regarding some preprocessing steps like bandpass-filtering, other steps like the impact of the reference electrode are still under debate, as the paper by Billings et al. (2021) in this special issue shows. Beyond preprocessing, if one wishes to make inferences on spatial dynamics, including connectivity between brain areas (as opposed to “connectivity” between electrodes), the choice of algorithm that produces the brain regions’, or source, activity often depends on the research question. It is our impression that a definite standardization is not (yet) possible. Still, there is an alternative to manually adjusting parameters, which is using automated pipelines—as described in the paper by Bhutada et al. (2021)—that allows objective assessment of data quality.

## Models that Bridge Spatial and Temporal Scales

Standardization will improve data quality and reproducibility in M/EEG research, but it will not abolish the limitations inherent in M/EEG signals. In order to go beyond these limitations, combining M/EEG with other neuroimaging techniques that have higher signal to noise-ratio and higher spatial resolution is an increasingly popular approach. An example can be found in the paper by Qin et al. (2021) in this special issue. However, in order to really integrate different neuroimaging methods—and “integration” here is meant in contrast to a mere “parallel processing” and subsequent comparison—we have to understand how the neural signals acquired by different techniques are underpinned by the same electrical or neurophysiological activity of the brain.

In the computational neuroscientist’s Utopian future, models mimic brain activity starting at the cellular level, while at the same time allowing for a direct validation with empirical M/EEG data. Indeed, the dynamics of local circuits and even single cells play a major role in understanding oscillations and other wave-related phenomena that are prominent features of M/EEG activity, as is shown in the papers by Saponati et al. (2021) and Hutt and Lefebvre (2021) as well as the opinion paper by Orczyk and Kajikawa (2021). It is also important to understand though that such detail is neither always necessary nor practical, especially when one wants to model global dynamics of the brain, as is done in the papers by Allouch et al. (2021) and Byrne et al. (2021): useful models reside on all spatial scales spanning from the micro- to the macroscopic level (Glomb et al. 2021).

## The Road Ahead: EEG Biomarkers and Human Behavior

Models link behavior to brain states that are generally impossible to observe, at least in humans. They do so by comparing empirical data to simulated data generated by the model on the basis of non-observable brain states (the parameters of the model). This point of view makes it clear how important it is to combine empirical data with modelling work. We have put emphasis on this point in our special issue—most papers contained in this collection use empirical data in some way.

Two papers in particular use data from auditory tasks. One, by Kohl et al. (2021), links a microscopic model of the canonical neocortical circuit to large-scale brain dynamics measured by M/EEG. Another, by Sancristóbal et al. (2021), links EEG power to reaction times. This way, these two papers exemplify how models can be used to link model parameters to behavior.

Without a model, one is left with correlations between measured data and behavior. However, with a model, one can exploit causal relationships to make predictions. These predictions can concern changes in the brain that underlie certain disorders. This kind of prediction is not only useful to basic science for understanding disease mechanisms, but it can lead to interpretable clinical biomarkers that are not only statistically related to a condition, but that tell us where in the brain what kind of change has occurred. Models also allow to characterize individuals by assigning them a unique set of parameter values. Individualized models are already a big topic in neuroscience, see e.g. The Virtual Brain^2^. The goal is to generate predictions for the individual, for example regarding disease progression or likely response to different kinds of medication.

If this individualized link between models and behavior can be achieved, M/EEG models will have fulfilled their potential to provide added value for doctors and scientists in their effort to understand the human brain.

## Footnotes

1 https://sites.google.com/view/ocns-2019-eeg-comp-model/home

2 www.thevirtualbrain.org
